# Urinary 6-sulphatoxymelatonin levels in patients with senile cataracts

**DOI:** 10.1186/1471-2415-13-46

**Published:** 2013-09-22

**Authors:** Muberra Akdogan, Yasemin U Budak, Kagan Huysal

**Affiliations:** 1 Department of Ophthalmology, Sevket Yilmaz Education and Training Hospital, Bursa, Turkey; 2 Department of Clinical Laboratory, Sevket Yilmaz Education and Research Hospital, Bursa, Turkey; 3 Department of Clinical Laboratory, Yüksek İhtisas Education and Research Hospital, Bursa, Turkey

**Keywords:** Senile cataract, 6-sulphatoxymelatonin, Antioxidant

## Abstract

**Background:**

The antioxidant melatonin effectively scavenges highly toxic hydroxyl radicals. Decreases in circulating melatonin levels have been reported in patients with diseases that become more serious with advancing age. The purpose of the present study was to explore the relationship between circulatory melatonin level and the extent of senile cataracts. To this end, we assessed the urinary excretion levels of 6-sulphatoxymelatonin (aMTS6), a major metabolite of melatonin.

**Methods:**

A total of 22 patients (aged 64 ± 7 years; 12 males and 10 females) with senile cataracts and 22 healthy controls (aged 61 ± 8 years, 12 males and 10 females) were studied. aMTS6 urine levels were measured using commercial ELISA kits. Each aMTS6 level was expressed as [aMTS6] (in ng)/[mg] creatinine. As the data were not normally distributed, the Mann–Whitney *U*-test was employed to assess the statistical validity of the difference observed.

**Results:**

The aMT6 level in nocturnal urine was 17.87 ± 14.43 ng aMTS6/mg creatinine (mean ± SD) in senile cataract patients; this was 76% of the level measured in age- and gender-matched controls (23.28 ± 16.27 ng aMTS6/mg creatinine). This difference in nocturnal urine aMTS6 level between senile cataract patients and controls was not statistically significant (p = 0.358).

**Conclusion:**

The urinary aMTS6 level did not differ between subjects with and without senile cataracts.

## Background

As is true of many other tissues, the normal human eye undergoes several structural changes with advancing age [1]. Most of the described anatomic and physiological changes are associated with gradual functional declines, such that the quality of vision deteriorates over time. Degenerative eye changes that progress more rapidly in some aging subjects than in others are associated with development of a variety of age-related ocular diseases including senile cataract formation and age-related macular degeneration (AMD) [2,3]; these conditions are the major causes of blindness worldwide [4].

The pathophysiologies of age-related ocular diseases are complex and remain poorly understood. Oxidative stress, associated with cellular damage caused by reactive oxygen intermediates (ROIs), has been implicated in the development of both AMD [5-8] and cataracts [5,9]. ROIs, which include free radicals, hydrogen peroxide, and singlet oxygen, are primarily generated as toxic byproducts of various cellular O2-consuming processes [10,11]. The retina is particularly susceptible to oxidative stress because this tissue is characterized by high oxygen consumption, has a high proportion of polyunsaturated fatty acids, and is regularly exposed to bright light [11]. Together, these factors create a chronic oxidative burden that can damage retinal proteins, DNA, and lipids [11]. Under normal conditions, the antioxidant defense systems of the body can easily neutralize free radicals produced by toxic reactions. However, although the extent of oxidative stress increases with age, the ability of cells to detoxify reactive oxygen intermediates deteriorates as subjects age. Together, deterioration of the capacity to detoxify such materials, and the cumulative effects of oxidative stress, have been proposed to constitute a local insult to lens proteins in the ageing eye; this is considered to be a major cause of cataract formation [12-14].

Melatonin (N-acetyl-5-methoxy-tryptamine), the principal secretory product of the pineal gland, is a most effective antioxidant that scavenges highly toxic hydroxyl radicals [15-19]. Endogenous melatonin production falls dramatically with age [20], and decreases in circulating melatonin levels have been reported in patients with age-related ocular diseases [21].

The purpose of the present study was to explore a possible relationship between circulatory melatonin levels and development of senile cataracts via assessment of 6-sulphatoxymelatonin (aMT6) excretion in first-morning urine samples, which is an accurate indicator of peak plasma melatonin concentrations during the previous night [22].

## Methods

The present case–control prospective study involved non-institutionalized subjects aged 60 years or more. All experiments were carried out in accordance with the tenets of the Declaration of Helsinki (1989) of the World Medical Association. Our study was approved by the Bursa Regional Ethics Committee and all subjects provided written informed consent.

All subjects in the case and control groups were examined by an expert ophthalmologist. Routine ophthalmic examinations including determination of visual acuity, slit lamp examination, ophthalmoscopy, and intraocular pressure measurement, were performed. A cataract was diagnosed when lens opacity was advanced to an extent that impaired vision. Controls were cataract-free subjects who were individually matched with cases on age and sex.

Inclusion criteria were the presence any type of senile cataract (posterior subcapsular, cortical, nuclear or mixed type) from grade I to III in an otherwise normal eye in patients older than 60 years. Any patient with secondary cataracts was excluded. Thus, those with cataracts caused by trauma, diabetes, and other known causes, did not participate in the study.

Subjects with a history of current or previous liver or kidney disease were excluded, as were shift workers, those with sleep disorders, those who had just experienced a long-distance flight, and those who had taken melatonin orally within the prior 2 weeks.

The aMTS6 levels in nocturnal urine specimens were determined in duplicate using a commercial competitive enzyme-linked immunosorbent assay (ELISA) kit from DRG Instruments GmbH (Marburg, Germany). Fifty microliters of 1:51 dilutions of urine samples were added to 50 μl of a solution of a melatonin sulfate peroxidase-conjugate, and then 50 μl of an anti-melatonin rabbit antibody solution was added. After incubation for 2 h at room temperature in an orbital shaker (500 rpm), all samples were washed four times with 250 μl of phosphate buffer and were next mixed with 100 μl of a tetramethylbenzidine/hydrogen peroxide solution (1:31, v/v). These mixtures were incubated for a further 30 min at room temperature on an orbital shaker. One hundred μl of 1 M sulfuric acid was subsequently added as the stopping solution. Absorption levels at 450 nm were measured within 1 h and the amount of aMTS6 in each urine specimen was calculated following the manufacturer’s instructions. The interassay coefficients of variation were 8.2% and 5.8% when 10 control samples with 14.5–62 aMTS ng/ml were assayed on two occasions.

All aMTS6 levels were standardized to those of creatinine to control for differences caused by variations in urine dilution. Urine creatinine levels were assayed using a commercial automated clinical chemistry system (Dimension RxL; Siemens Healthcare Diagnostics Inc., Newark, DE). In each instance, the urine aMTS6 level was divided by the urine creatinine concentration to obtain a normalized value for the urine aMTS6 level, expressed as ng aMTS6/mg creatinine.

### Statistical analysis

All statistical tests were conducted with the aid of SPSS for Windows version 13.0 (SPSS Inc., Chicago, IL). Data are presented either as percentages or as means ± SDs. The Mann–Whitney *U* test was used, when appropriate, to assess the significance of observed differences between the two groups. A p value of ≤0.05 was considered to be significant.

## Results

**Figure 1:**
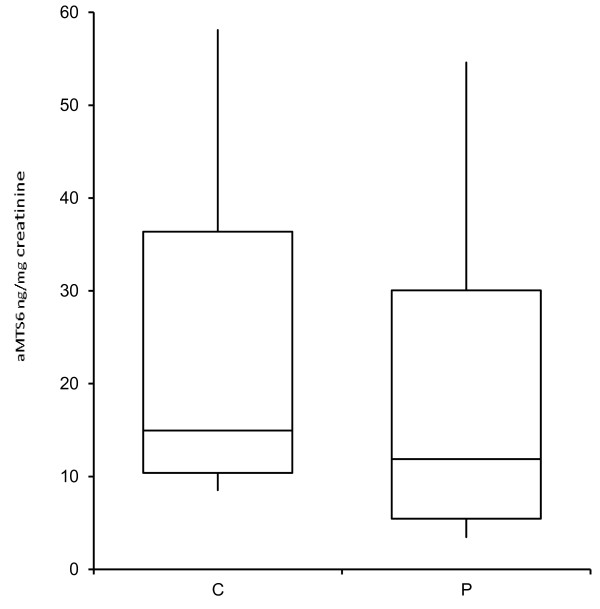
**Urinary ng MTS6/mg creatinine levels in patients with senile cataract (P), and matched control subjects (C).** The center lines represent the median values.

Twenty-two senile cataract patients (aged 64 ± 7 years; 12 males and 10 females), and 22 normal subjects (aged 61 ± 8 years; 12 males and 10 females), were studied (Table 1). The aMTS6 level in nocturnal urine was 17.87 ± 14.43 ng/mg creatinine (mean ± SD) in senile cataract patients; this was 77% of the value observed in age- and gender-matched controls (23.28 ± 16.27 ng/mg creatinine (min: 8.52-max:58.10 ng/mg creatinine)) (Figure 1). This difference was not statistically significant (p = 0.358).

**Table 1:** Demographical and laboratory data of senile cataract patients and controls

	**Senile cataract**	**Control**
Age	64 ± 7	61 ± 8
Gender (male/female)	12/10	12/10
aMTS6 (ng/mg creatinine)	17.87 ± 14.43 (3.48-54.61)*	23.28 ± 16.27 (8.52-58.10)*

*Numbers in parentheses indicate minimum and maximum values.

## Discussion

We explored whether melatonin levels differed between patients with age-related cataracts and normal subjects by measuring the levels of aMTS6, a major metabolite of melatonin. The urinary excretion level of aMTS6 has been previously shown to be a reliable surrogate of the blood melatonin level, reflecting nocturnal synthesis of melatonin by the pineal gland [23]. High correlations between 24-h plasma and serum melatonin levels, on the one hand, and urinary melatonin levels, on the other, have been reported previously [24-27].

Although the pathogenesis of senile cataracts remains poorly understood, many studies have suggested that genetic factors and oxidative stress are both of major importance in the development of the condition [28]. Hundreds of publications have described the free-radical- scavenging and antioxidant actions of melatonin [29]. Melatonin levels in patients with AMD have been suggested to be lower than in aged subjects without AMD, because the former patients show greater decreases in nocturnal urine aMTS6 levels than do the latter. Thus, AMD may be associated with a greater decrease in melatonin levels than is characteristic of those who age normally, and a melatonin deficiency may influence development of AMD [30].

The melatonin levels of senile cataract patients have not been previously reported. To the best of our knowledge, the present study is the first to compare urinary aMTS6 levels in senile cataract patients with those in age-matched controls. Our initial hypothesis was that senile cataract patients would have lower aMTS6 levels than did normal subjects. Although the level of urinary aMTS6 in senile cataract patients was 33% lower than in age-matched controls, the difference was not statistically significant.

We found no relationship between urinary aMTS6 levels and development of senile cataracts. It is widely accepted that melatonin production by the pineal gland declines progressively with age [31]. Therefore, in elderly subjects, the melatonin level is much less than that of young individuals [32]. Melatonin is produced in the pineal gland but also in many other tissues and organs; such extrapineal production is much greater than that of the pineal gland [33-35]. However, even the most active peripheral sources of melatonin (including the retina) appear to contribute to only a limited extent to systemic hormone levels; the level of local catabolism appears to be high. Melatonin can be produced by ocular cells, pinealocytes, retinal photoreceptors, and ciliary epithelial cells [36-38], all of which operate under the direct control of a circadian clock [39]. Indeed, previous studies have shown that retinal melatonin may possibly act as a free-radical scavenger within photoreceptors [40,41]. Local production of melatonin may cause the melatonin concentration surrounding photoreceptors to be relatively high, protecting such cells from oxidative insult either via exertion of a direct antioxidant activity or via activation of melatonin receptors. The level of melatonin produced in the eye is far less than that produced in the pineal body [36,37,41]. It is widely accepted that blood melatonin is derived exclusively from the pineal gland in mammals [4,36,38]. Therefore, the circulating melatonin level is only one of several factors determining melatonin levels in the eye. Because any association between the melatonin levels of the blood and the lens is poorly understood, it remains unclear whether existing biochemical data (largely derived from measurements performed on blood) accurately reflect lens melatonin levels. Therefore, we cannot conclude that ocular melatonin levels differed between our senile cataract patients and the control group.

The limitations of the present study are that the number of cases was limited, possibly compromising the statistical power of the study; and also that the standard deviations in mean urinary melatonin concentrations were large.

## Conclusion

We found that the urinary aMTS6 level did not appear to differ between those with and without senile cataracts. Our findings do not support the hypothesis that age-related reductions in the efficacy of antioxidant defense systems trigger biochemical cascades causing age-related cataract formation. However, future studies including larger sample sizes are warranted to confirm the validity of this observation, as well as to reveal the exact role of melatonin in senile cataract formation.

## Competing interests

The authors declare that they have no competing interests.

## Authors’ contributions

YB contributed to the study design and did critical revision of the manuscript for important intellectual content. MA participated in the eye examinations. KH performed the analysis of the data. All authors read and approved the final manuscript.

## Pre-publication history

The pre-publication history for this paper can be accessed here:

http://www.biomedcentral.com/1471-2415/13/46/prepub
